# Investigation of *ginkgo biloba* leave extracts as corrosion and Oil field microorganism inhibitors

**DOI:** 10.1186/1752-153X-7-83

**Published:** 2013-05-07

**Authors:** Gang Chen, Min Zhang, Jingrui Zhao, Rui Zhou, Zuchao Meng, Jie Zhang

**Affiliations:** 1 College of Chemistry and Chemical Engineering, Xi’an Shiyou University, Xi’an Shaanxi, 710065, People’s Republic of China; 2 Shannxi Hai’an Industry Co., LTD, Xi’an, 710065, People’s Republic of China

**Keywords:** *Ginkgo biloba*, Extract, Inhibition efficiency, Oil field microorganism

## Abstract

*Ginkgo biloba* (Ginkgoaceae), originating from China, now distributes all over the world. Wide application of *Ginkgo biloba* extracts is determined by the main active substances, flavonoids and terpenoids, which indicates its extracts suitable to be used as an effective corrosion inhibitor. The extracts of *Ginkgo biloba* leave have been investigated on the corrosion inhibition of Q235A steel with weight loss and potentiodynamic polarisation techniques. The inhibition efficiency of the extracts varies with extract concentration. The extracts inhibit corrosion mainly by adsorption mechanism. Potentiodynamic polarisation studies show that extracts are mixed type inhibitors. The antibacterial activity of the extracts against oil field microorganism (SRB, IB and TGB) was also investigated.

## Background

To prove the crude oil recovery, acidification of the low permeable reservoirs is one of the efficient operations, during which hydrochloric acidic fluid is pumped into wells to etch the fracture walls irregularly and create highly conductive channels [1]. While it is also a great challenge for the metal instruments involved in the acidification, and there is a need to improve the resistance properties of the steel against such corrosion in acidic media. Great attention has been paid to prolonging the lifetime, in which the use of corrosion inhibitors is considered as the most effective method against such acid attack [2,3]. Functional electronegative groups and *p*-electron in conjugated double or triple bonds, are the major adsorption centers. So the organic compounds, containing heteroatoms, such as sulfur, phosphorus, nitrogen and oxygen, together with aromatic rings, are efficient as corrosion inhibitors [4-7].

**Figure 1:**
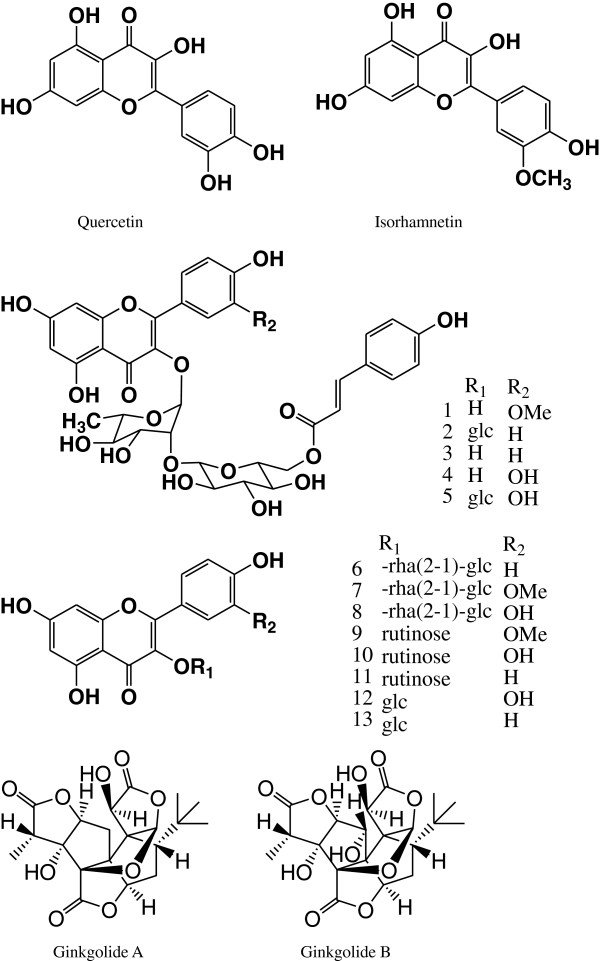
**Structures of flavonoids and terpenoids found in *Ginkgo biloba*.**

Although many compounds have been synthesized for the corrosion inhibition, there is a need to develop green and eco-friendly corrosion inhibitors with low cost. In fact, many plants’ extracts have been screened as corrosion inhibiters for mild steel [8-10], and the tremendous plant resources in Qin-ling and Ba-shan Mountain of Shannxi Province provide the guarantee for the natural material exploration. *Ginkgo biloba* (Ginkgoaceae), originating from China, was first introduced to Europe in the 18th century, and now distributes all over the world. Wide application of *Ginkgo biloba* extracts is determined by the main active substances: flavonoids and terpenoids (Figure 1) and has shown antioxidant activity in preliminary DPPH assays [11-14], which indicates the *Ginkgo biloba* extracts suitable to be used as an effective corrosion inhibitor. The aim of this work is to investigate the inhibitory action of *Ginkgo biloba* extract for the corrosion of mild steel of oil field.

## Experimental

### Materials and methods

*Ginkgo biloba* leave were washed and dried under 60°C and was shattered into powder, and the powder was heated with water or alcohol for 4 h. Then a yellow aqueous extract was filtered to yield dry extract after removal of the solvent. The extract yield from the water and alcohol were named as WE and AE, and extraction rate of water and alcohol is 29.8% and 18.7% respectively.

### Gravimetric measurements

The corrosion tests were performed on Q235A with a composition (in wt.%) C: 0.22, P: 0.045, Si: 0.35, S: 0.05, Mn: 1.40, and Fe balance. The electrolyte solution was 1M HCl, prepared from analytical grade 38% HCl and distilled water. The concentrations of pomegranate husk extract were employed as 10 mg/L, 100 mg/L and 1000 mg/L. All tests have been performed in deaerated solutions and at 60 ± 0.5°C. The gravimetric tests were carried out according to the People’s Republic of China Standard of Petroleum and Natural Gas Industry (Evaluation method for behavior of corrosion inhibitor for produced water of oilfield, SY/T5273-2000) with a few modifications. Each test was done with three specimens at the same time to give reproducible results.

### Electrochemical measurements

The electrodes were mechanically abraded with a series of emery papers (800 and 1200 grad). Then it was rinsed in acetone and double distilled water before their immersion in experimental solution. Electrochemical measurements were conducted in a conventional three-electrode thermostated cell. The electrode was inserted in Teflon tube and isolated with polyester so that only its section (0.5 cm^2^) was allowed to contact the aggressive solutions. A platinum disk as counter electrode and standard calomel electrode (SCE) as the reference electrode have been used in the electrochemical studies.

The potentiodynamic curves were recorded using a CS350 system connected to a personal computer. The working electrode was first immersed into the test solution for 60 min to establish a steady state open circuit potential. After measuring the open circuit, potential dynamic polarisation curves were obtained with a scan rate of 0.5 mV/s. Corrosion rates (corrosion current densities) were obtained from the polarisation curves by linear extrapolation of the anodic and cathodic branches of the Tafel plots at points 100 mV more positive and more negative than the corrosion potential (Ecorr).

CS350 electrochemical workstation hardware parameters:

Potentiostat potential control: ± 10 V; Current Control Range: ± 2.0A; Potential control precision: 0.1% × full scale reading ± 1 mV; Current control accuracy: 0.1% × full scale reading; Potential resolution: 10 μV (>100 Hz), 2 μV (< 10 Hz); Current resolution: < 10pA; Potential rise time: < 1 μS (< 10 mA), < 10 μS (< 2A); Auxiliary 24-bit data acquisition-10 KHz, 20bit-1 KHz; Reference electrode input impedance: 1012 ohms || 20pF; Current range 2A- 00 nA, a total of 8 files; Tank pressure: 21V; CV and LSV scan rate: 0.01-20000 mV/s; CA and CC pulse width: 0.0001-1000 s; Potential scan potential incremental: 0.1 mV-1V/mS; SWV frequency: 0.001-100 KHz; DPV and NPV pulse width: 0.0001-1000 s; AD data acquisition: 16 bits-1 MHz, 24bit-100 Hz; Minimum potential increment CV: 0.075 mV.

### Microbiological monitoring

Viable counts of SRB, TGB and FB were determined according to the Standard of Petroleum and Natural Gas Industry of People’s Republic of China (Method of SY/T 5890–1993, The national method of the bactericidal agent’s performance). The produced water containing the three kinds of bacteria was gathered from Zichang Oilfield Factory, Yanchang Oilfield.

## Findings

### Inhibitor properties and mechanism

Q235A (A3) steel is widely used in the gas and oil field, and it is easily eroded in the presence of high concentration of HCl under high temperature. The use of corrosion inhibitors, such as imidazoline, Mannich base, Schiff base and some other heterocyclic compounds, is considered as the most effective method for the protection against such acid attack, but the concentration and the cost is too high to be accepted.

**Figure 2:**
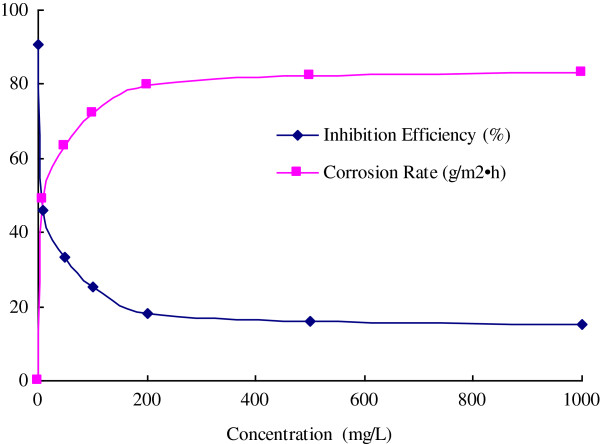
The inhibition efficiency and corrosion rate of WE.

**Figure 3:**
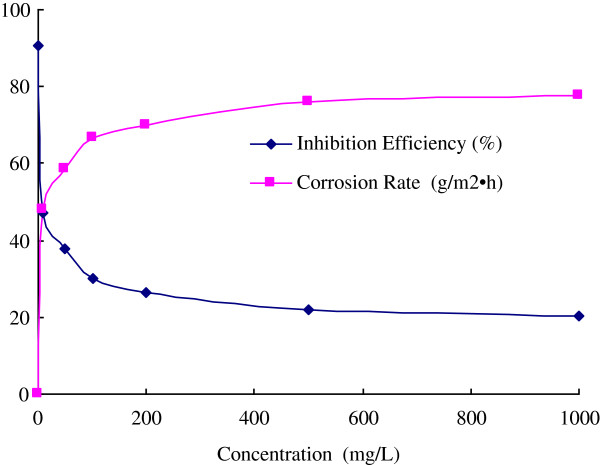
The inhibition efficiency and corrosion rate of AE.

Developing natural products as oilfield chemicals is a direct way to find green and eco-friendly materials with low cost. Based on the various and large quantity of local resources of Qin-ling and Ba-shan Mountain, several plants have been investigated for the application in oilfield chemistry in our research work. The inhibition efficiency (IE) of extract of *Ginkgo biloba* leave was investigated with the concentration range from 10 mg/L to 1000 mg/L in 1 M HCl, and the change of IE (%) and corrosion rate (g/m^2^ · h) with the inhibitor concentration were summarized in Figures 2 and 3. From the table, it was found that the extract can inhibit the corrosion with different efficiency under different concentrations, and the IE increases with the concentration. For WE, the IEs reach to 72.0% and 83.2% with the concentration of 100 mg/L and 1000 mg/L respectively, while the IEs of AE only reach to 66.7% and 77.7% respectively.

### Tafel polarisation measurements

**Figure 4:**
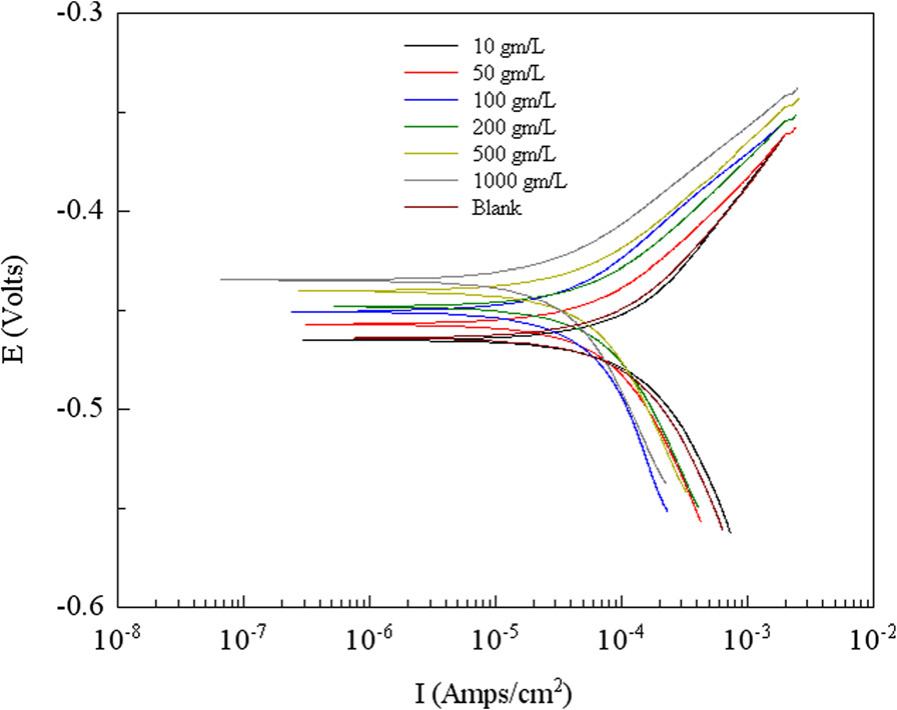
Typical polarization curves for corrosion of Q235A steel in 1M HCl in the absence and presence of different concentrations of WE.

**Figure 5:**
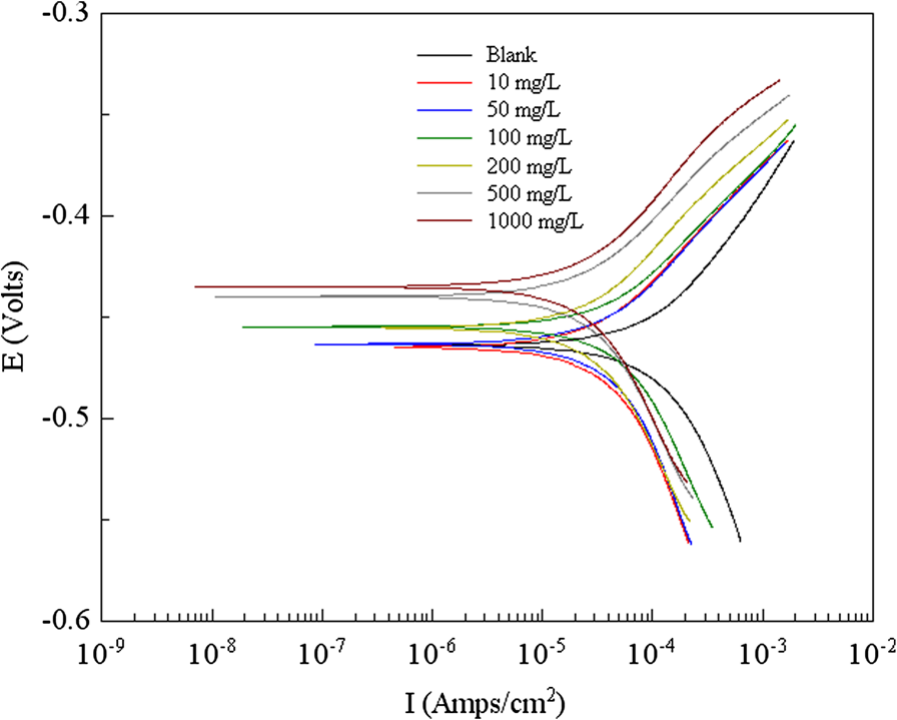
Typical polarization curves for corrosion of Q235A steel in 1M HCl in the absence and presence of different concentrations of AE.

Both anodic and cathodic polarization curves for a carbon steel electrode in 1.0 M HCl at various concentrations of the WE and AE at 298 K are given in Figures 4 and 5. The electrochemical corrosion kinetic parameters, i.e., corrosion potential (Ecorr), cathodic and anodic Tafel slopes (βa, βc) and corrosion current density icorr obtained by extrapolation of the Tafel lines are included in Table 1. The calculated inhibition efficiency, IE (%) is also calculated from the following equation:

(1) $$E\left(\%\right)=\frac{I_{\mathrm{corr}}-I_{\mathrm{corr}\left(i\right)}}{I_{\mathrm{corr}}}\times 100$$

**Table 1:** Potentiodynamic polarization parameters for the corrosion of the Q230A steel in 1M HCl in absence and presence of different concentrations of the extracts

**Extract**	**Concentration**	**−Ecorr**	**Icorr**	**βa**	**βc**	**Corrosion rate**	**IE (%)**
	**(mg/L)**	**(mV)**	**(μA/cm**^**2**^**)**	**(mV/dec)**	**(mV/dec)**	**(mm/a)**	
--	--	0.46083	151.440	90.43	155.08	1.7753	--
WE	10	0.47503	131.180	86.83	160.49	1.5430	13.1
WE	50	0.45705	96.410	72.35	151.56	1.1340	36.1
WE	100	0.45056	57.454	65.77	161.94	0.6758	61.9
WE	200	0.4455	55.195	59.85	143.79	0.6492	63.4
WE	500	0.43980	34.272	55.18	134.99	0.4031	77.3
WE	1,000	0.42657	33.219	52.64	128.09	0.3907	78.0
AE	10	0.45347	111.570	77.58	152.17	1.3123	26.1
AE	50	0.45128	85.704	67.94	129.73	1.0081	43.2
AE	100	0.45489	54.511	65.70	121.36	0.6412	63.9
AE	200	0.45549	31.770	64.64	113.19	0.3737	78.9
AE	500	0.43987	29.917	62.11	112.71	0.3525	80.1
AE	1,000	0.42490	29.194	68.59	116.21	0.3434	80.7

Where Icorr and Icorr(i) are corrosion current densities obtained in the absence and presence of inhibitors, respectively. It can be seen that the corrosion rate is decreased and inhibition efficiency IE (%) is increased by increasing inhibitor concentration [15]. The extract causes changes in the anodic, cathodic Tafel slopes and the Ecorr values in the presence of different concentrations, suggesting that these compounds behave as mixed-type (anodic/cathodic) inhibitors [16-18]. Increasing inhibition efficiencies with increasing concentrations of the extract shows that the inhibition actions are due to its adsorption on the steel surface [19,20]. The difference in inhibition efficiency between WE and AE may be due to the effect of the high water-soluble part, which includes the glycoside and some polysaccharide. The inhibition efficiencies obtained from potentiodynamic polarization were different from those calculated from weight-loss measurements, which may be attributable to the fact that the weight-loss method gives average corrosion rate, whereas electrochemical method gives instantaneous corrosion rates. These variations may also arise because of the difference in the time required to form an adsorbed layer of inhibitors on metal surface that can inhibit corrosion [21,22].

### Mechanism of corrosion inhibition

It is well recognized that organic inhibitor molecules set up their inhibition action via the adsorption onto the metal/solution interface. The adsorption process is affected by the chemical structures of the inhibitors, the nature and charged surface of the metal and the distribution of charge over the whole inhibitor molecule. The presence of N, O, S atoms and conjugated double bonds in the organic structures makes the formation of *p*–*d* bonds resulting from overlap of *p*-electrons to the 3*d* vacant orbital of iron atoms, which enhances the adsorption of the compounds on the metal surface [23-25]. In general, organic inhibitor molecules may be adsorbed on the metal surface in one or more of the following ways [26,27]:

(a) electrostatic interaction between the charged molecules and the charged metal;

(b) interaction of unshared electron pairs in the molecule with the metal;

(c) interaction of p-electrons with the metal;

(d) a combination of types (a–c).

**Figure 6:**
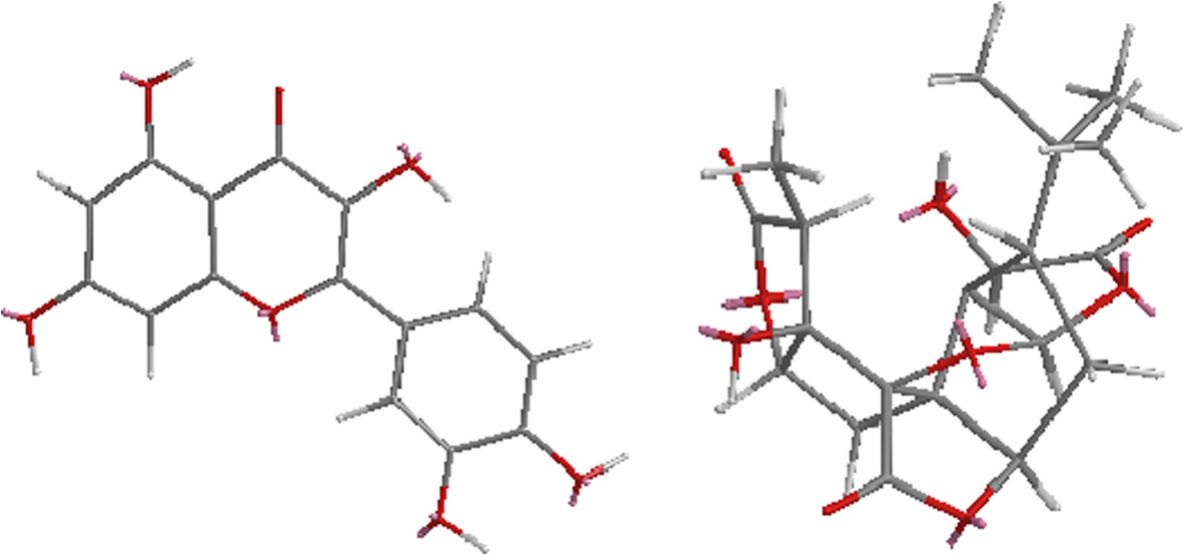
The steady conformation of Quercetin and Ginkgolide A.

But owing to the complex nature of adsorption and inhibition of a given inhibitor, it is impossible for single adsorption mode between inhibitor and metal surface. The structures of phenols and terpenoids in *Ginkgo biloba* leave were shown in Figure 1, and the steady conformation of Quercetin and Ginkgolide A were shown in Figure 6, which were simulate by a minimize energy of MM2 in Chem 3D, and the *p*-electrons of the hydroxyl groups and ether groups were colored in pink.

**Figure 7:**
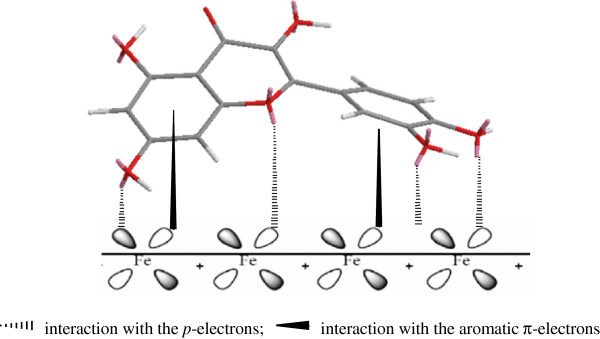
The absorption of Quercetin on the steel surface by coordination.

**Figure 8:**
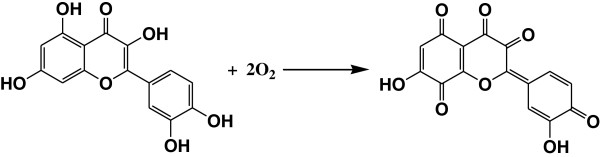
**The oxidation of Quercetin by O**_**2**_**.**

The inhibition efficiency afforded by Quercetin may be attributed to the presence of electron rich phenol groups and aromatic rings, while Ginkgolide A may be attributed to the presence of electron rich O. The possible reaction centers are unshared electron pair of hetero-atoms and/or *p*-electrons of aromatic ring. Generally, Quercetin can absorb on the mild steel surface on the basis of donor–acceptor interactions between *p*-electrons of the O and aromatic ring and vacant *d* orbitals of surface iron. The schematic illustration of different modes of adsorption on metal is shown in Figure 7. Besides, the flavonoids are high reductive and can be oxidized to benzoquinone by the O_2_ resolved in the solution, which can inhibit the oxygen-adsorption corrosion (as shown in Figure 8) [28,29].

### Antibacterial activity against oil field microorganism

**Figure 9:**
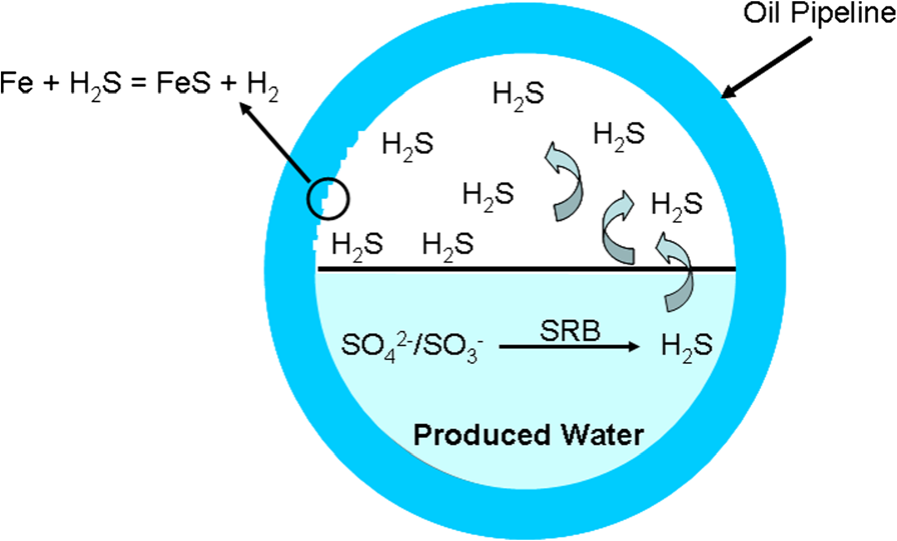
Mechanism of the microbial corrosion of oil pipelines.

Produced water is a consequence of oil field exploitation by waterflood or steam injection or having an aquifer linking to the reservoir. The most usual disposal way for the high-volumed produced water is to re-inject it to the well after treatment, which will meet some requirements imposed by environmental and exploitation regulations [30], among which microbiologically influenced corrosion (MIC) is an example. MIC, mainly caused by the growth of such oil field microorganism as sulfate reducing bacteria (SRB), iron bacteria (IB) and total general bacteria (TGB) in oil pipelines, is considered as a major problem for water treatment in the oil field [31]. MIC can result in different types of attack, such as pitting, crevices, dealloying and erosion in pipelines (as shown in Figure 9) [32]. Even more serious, the interaction of IB, SRB and TGB can accelerate the corrosion rate, in other word, the corrosion in the mixture of IB, SRB and TGB was more serious than in a single microbial system. Based on this case, different treatment system to inhibit corrosion should be considered, among which using bactericide has received the greatest acceptance. Currently, oxidizer, aldehyde, quatemary ammonium salt and heterocycle compounds, such as Cl_2_, ClO_2_, formaldehyde, pentane-1,5-dial, trichloroisocyanuric acid (TCCA) and ect., have been used as bactericides [33,34], but the toxicity and oxidation tests have been conducted on a limited selection.

Since the structures of the main composition in *Ginkgo biloba* leave have been determined in Figure 1, and the flavonoids can combine with proteins and show some antibacterial activities [35], the extracts are anticipated to be bactericides for oil field bacteria. In the following work, the antifungal activity of these extracts against oil field microorganism was tested under the concentrations of 500 mg/L and 1,000 mg/L, and the results are summarized in Table 2. From the table, it is apparent that extracts are highly antifungal active against the three microorganisms under the concentration of 1,000 mg/L. As the concentration of the extracts is reduced to 500 mg/L, the inhibitions are still potent for most of the cases with slightly higher microbiotic concentrations. But the inhibitions of WE for TGB and AE for IB and TGB depressed obviously.

**Table 2:** **The antifungal activity of*Ginkgo biloba*leave extract against oil field MIC**

**Extract**	**Concentration mg/L**	**Microbiotic concentration/mL**		
		**SRB**	**IB**	**TGB**
—	—	110.0	110.0	110.0
WE	500	2.0	1.3	20.0
	1,000	2.0	2.0	2.0
AE	500	2.5	110.0	25.0
	1,000	2.5	6.0	2.5

## Conclusions

The water and alcohol extracts of *Ginkgo biloba* leave showed moderate to high effective inhibition in the range 10 to 1,000 mg/L in 1 M HCl at 60°C, and the highest inhibition of 83.2% was obtained by using WE solution of 1,000 mg/L. The extracts mainly inhibit corrosion by adsorption mechanism. Tafel polarisation measurements indicate the extracts behave as mixed type inhibitor. Investigation of the antibacterial activity against oil field microorganism showed the extracts can inhibit SRB, IB and TGB with high efficiency under 1,000 mg/L, which makes extracts potential to be used as bifunctional oil field chemicals.

## Competing interests

The authors declare that they have no competing interests.

## Authors’ contributions

GC has conceived the study, formulated the research idea and prepared the manuscript draft version, MZ, RZ, ZM carried out the corrosion inhibition experiments, J Zhang carried out the Microbiological monitoring, and J Zhao participated in its design and coordination. All authors have read and approved the final manuscript.
